# Steroid Receptor RNA Activator (SRA) Modification by the Human Pseudouridine Synthase 1 (hPus1p): RNA Binding, Activity, and Atomic Model

**DOI:** 10.1371/journal.pone.0094610

**Published:** 2014-04-10

**Authors:** Tiphaine Huet, François-Alexandre Miannay, Jeffrey R. Patton, Stéphane Thore

**Affiliations:** 1 Department of Molecular Biology, University of Geneva, Sciences III, Geneva, Switzerland; 2 Department of Physical Chemistry, University of Geneva, Sciences II, Geneva, Switzerland; 3 Department of Pathology, Microbiology and Immunology, University of South Carolina, School of Medicine, Columbia, South Carolina, United States of America; MRC National Institute for Medical Research, United Kingdom

## Abstract

The most abundant of the modified nucleosides, and once considered as the “fifth” nucleotide in RNA, is pseudouridine, which results from the action of pseudouridine synthases. Recently, the mammalian pseudouridine synthase 1 (hPus1p) has been reported to modulate class I and class II nuclear receptor responses through its ability to modify the Steroid receptor RNA Activator (SRA). These findings highlight a new level of regulation in nuclear receptor (NR)-mediated transcriptional responses. We have characterised the RNA association and activity of the human Pus1p enzyme with its unusual SRA substrate. We validate that the minimal RNA fragment within SRA, named H7, is necessary for both the association and modification by hPus1p. Furthermore, we have determined the crystal structure of the catalytic domain of hPus1p at 2.0 Å resolution, alone and in a complex with several molecules present during crystallisation. This model shows an extended C-terminal helix specifically found in the eukaryotic protein, which may prevent the enzyme from forming a homodimer, both in the crystal lattice and in solution. Our biochemical and structural data help to understand the hPus1p active site architecture, and detail its particular requirements with regard to one of its nuclear substrates, the non-coding RNA SRA.

## Introduction

Pseudouridine is a modified uridine known to be essential for the function of most classes of non-coding RNAs (ncRNA) such as tRNAs, rRNAs, snoRNAs, or snRNAs [1], [2]. Pseudouridines are present in RNA from bacteria to mammals, and their synthesis is due to a protein family named pseudouridine synthases (PUS; [3]). Pseudouridine synthases are divided into six distinct families: TruA, TruB, TruD, RluA, RsuA, and Pus10, with the last one being present only in archaea and eukaryotes [4], [5]. Atomic models for various members of these families have been solved and show a conserved catalytic core despite very low sequence homology between them [5], [6], [7], [8], [9]. In addition, several secondary structure elements or entire domains are found around the structurally conserved core of particular members [5], [7], [10]. Humans have a variety of pseudouridine synthases, which act on diverse classes of ncRNAs. One of the first identified was the pseudouridine synthase 1 (hPus1p), which is a member of the TruA family despite their low sequence similarity (<20%;[11]). The hPus1p enzyme was identified in the late 1990′s on the basis of its sequence similarity with the homologous yeast enzyme. The eukaryotic Pus1 enzyme must localise in the mitochondria, the cytoplasm, and the nucleus based on the location of its identified substrates or partners [12], [13], [14]. More recently, the enzyme was shown to co-localise with particular nuclear receptors in the nucleus [12], [15], [16].

Although members within the PUS family do not exhibit extensive sequence homology, they share an enzymatic domain that presents a high degree of structural similarity [11]. The active site is located in between the two lobes of the catalytic core [6], [17], [18], [19], [20]. PUS enzymes are highly specific, capable of recognising their target uridine when embedded in a particular structural context, avoiding random uridine modification within RNA molecules. The hPus1p enzyme is no exception, although it appears to have a more relaxed sequence specificity compared to other pseudouridine synthases [21]. The TruA family is the most divergent compared to the other families [4]. The major sites of modification by the eukaryotic Pus1p enzyme are positions 27, 28, 34, and 36 within tRNAs [22], [23]. In addition, yeast Pus1 has been shown to modify U2 snRNA [24]. A few years ago, the Steroid receptor RNA Activator, a ncRNA emanating from the *SRA* gene, was characterised as a target of Pus1p [12], [15], [16]. Multiple sites within the SRA were shown to be subject to pseudouridine modification, although only U206 within the H7 element was identified unambiguously [15]. Lastly, the hPus1p enzyme is involved in the metabolic syndrome causing mitochondrial myopathy and sideroblastic anemia (MLASA; [25]).

We have characterised the catalytic domain of the hPus1p protein biochemically and structurally. A truncated protein has significant levels of activity towards a target tRNA and on the specific H7 element from the SRA, when compared to the full-length hPus1p enzyme. We also measured the affinity of the truncated form of hPus1p (ΔhPus1p) for various H7 SRA substrates, which correlates with the observed activities. We determined the structure of the catalytic domain of ΔhPus1p and the D146A mutant of this enzyme. We observe several molecules in the active site although their significance for the pseudouridine reaction remains unclear. Despite the low sequence-dependence shown for pseudouridylation by hPus1p, we suggest that the secondary structure of the substrate RNA is important for hPus1p-mediated activity. The atomic model of ΔhPus1p further demonstrates why the hPus1p enzyme binds to its RNA substrate as a monomer. The C-terminal end of hPus1p folds into two α-helices and these helices pack against the back of the hPus1p molecule, which prevents the dimerization observed in the bacterial homologue, TruA [6]. We propose that this C-terminal region is responsible for the atypical RNA substrate binding and activity of hPus1p.

## Materials and Methods

### Expression and purification

The fragment of hPus1p encompassing residues 83–394 (referred to as ΔhPus1p hereafter) was amplified by PCR using appropriate oligonucleotides from the hPus1p-containing plasmid obtained previously [22]. We subcloned this fragment, using restriction sites PciI and XhoI, into a modified pET vector for subsequent over-expression in *Escherichia coli*. The protein was expressed in *E. coli* BL21 (DE3) Star with an N-terminal 9Xhistidine tag for purification. Briefly, cells were grown in TB medium at 37°C, and subsequently incubated overnight at 18°C with 0.1 mM isopropyl thio-β-D-galactoside (IPTG). Cells were harvested by centrifugation at 4000×g, and resuspended in 20 mM HEPES (pH 7.0) containing 500 mM NaCl, 5% glycerol, 30 mM imidazole, 2 mM CHAPS, 1 mM DTT, DNase 1 (1 µg/ml), lysosyme (1 µg/ml), and a cocktail of protease inhibitors (phenylmethylsulphonyl fluoride 1 mM, leupeptin 2 µg/ml and pepstatin 2 µg/ml). Cells were lysed using an EmulsiFlex C3 (Avestin) homogenizer, and were clarified by centrifugation at 30,000×g for 30 minutes at 4°C. The protein was purified with an affinity chromatography step on a nickel-chelating resin (HisTrap, GE Healthcare), and eluted in the same buffer supplemented with 500 mM imidazole. The tag was cleaved with Tobacco Etch Virus (TEV) protease for 10 hours at 4°C. The cleaved sample was reloaded to the affinity column to eliminate the uncleaved protein, the tag, the protease, and remaining contaminants. The protein was applied to a gel filtration Superdex S75 16/60 column (GE Healthcare) pre-equilibrated with 20 mM HEPES (pH 7.0) containing 150 mM NaCl, 5 mM MgCl_2_ and 5 mM TCEP. The purity and homogeneity of the protein were assessed by SDS-PAGE. Full length (FL) hPus1p was expressed and purified in the same manner.

### Site directed mutagenesis

The D146A mutation was introduced to ΔhPus1p using the Quick Change mutagenesis kit (Stratagene) according to manufacturer instructions. The oligonucleotides used were:

5′CAGCGCTGCGCCCGGACAGCCAAGGGTGTGTCCGCAGCC and its reverse complement. The mutation was confirmed by sequence analysis. The D146A mutant version of ΔhPus1p was purified by methods described for ΔhPus1p.

### Crystallisation and structure determination

For crystallisation assays, proteins were concentrated to 7 mg/ml, and used for crystallisation after 2 days. We used the sitting drop technique for crystallisation, and mixed the protein with the reservoir solution in a 1∶1 ratio. Crystals of ΔhPus1p appeared after 2–3 days in a buffer containing 30% PEG 4000/glycerol, 0.1 M Bicine/TRIS (pH 8.5), 0.02 M amino acids mixture (0.02 M sodium L-glutamate, 0.02 M DL-alanine, 0.02 M glycine, 0.02 M DL-lysine HCl, 0.02 M DL-serine) at 4°C. The D146A ΔhPus1p mutant crystallised in the following buffer: 20% PEG 8000, 0.1 M TRIS (pH 8.5) at 4°C. Optimized crystallisation conditions are 28% PEG 3350/glycerol, 0.1 M Bicine/TRIS (pH 8.5), and 10% amino acids mixture for the ΔhPus1p and 17% PEG 8000, 0.1 M HEPES (pH 8.0) for the D146A mutant.

Crystals were flash cooled in liquid nitrogen after cryo-protection with the reservoir solutions enriched with 10% PEG 400 and 5% glycerol. Data collection from single crystals was performed at ID23-2 at the ESRF (Grenoble, France), and PXIII at the SLS (Villingen, Switzerland). About 20 data sets were collected from single crystals and co-crystals, with only slight variation in the cell parameters. Datasets were integrated and scaled using XDS [26]. Generally, crystals belong to space group P2_1_2_1_2_1_ or P22_1_2_1_ with two or one monomer(s) per asymmetric unit, respectively. Datasets suffered from anisotropy and pseudo-translational symmetry, which initially prevented us from solving the structure by molecular replacement. We finally solved the structure with PHASER [27] using Pus1p as a model (pdb id: 4ITS; [28]).

Alternative cycles of maximum likelihood refinement were performed with PHENIX [29], and model fitting was carried out with COOT [30]. Structures of ΔhPus1p and the ΔhPus1p D146A mutant were refined to 2.0 Å and 2.7 Å resolution, respectively. Crystallographic data are summarized in Table 1. Strong positive electron density peaks were observed next to the catalytic residue D146 in ΔhPus1p or the A146 residue in the mutated enzyme. Omit maps, calculated with only the protein models, were used to fit putative ligands found in the crystallisation condition (see Figure S5). Anisotropic scaling, bulk solvent correction, and TLS (Translation/Libration/Screw) restraints were used throughout the refinement procedure. Solvent molecules were added last in the unassigned peaks of the F_o_-F_c_ Fourier electron density map. The models show good stereochemistry with no residues in the disallowed region of the Ramachandran plot. The accession number for the coordinates of the structures of ΔhPus1p and D146A ΔhPus1p have been deposited with PDB codes 4NZ6 and 4NZ7, respectively.

**Table 1:** Data collection and refinement statistics.

	ΔhPus1p	ΔhPus1p D146A
Beamline	X06DA SLS	ID 23-2 ESRF microfocus
***Data processing***		
Resolution (Å)	50–2.0 (2.05–2.00)	50–2.7 (2.77–2.70)
Crystal space group	P2_1_2_1_2_1_	P22_1_2_1_
Cell parameters (Å)	a = 71.42; b = 75.12; c = 110.58	a = 39.45; b = 69.04; c = 116.78
Unique reflections	40510 (2984)	9281 (686)
R_meas_ (%)^a^	8.5 (55.8)	14.9 (71.9)
Mean redundancy	4.3 (4.2)	5.0 (5.1)
CC_1/2_ (%)	99.8 (82.9)	99.6 (88.5)
Completeness (%)	99.3 (98.8)	99.8 (100)
Mean I/σ (%)	16.4 (2.5)	13.8 (3.4)
***Refinement***		
Resolution (Å)	30–2.0	20–2.7
rmsd bond (Å)	0.002	0.005
rmsd angle (°)	0.633	0.863
R_cryst_ (%)^b^	18.40	18.33
R_free_ (%)^c^	22.41	23.81
*Number of atoms:*		
protein	4949	2419
Bound compounds	20	15
PEG	14	10
water	479	110
*Average B factors:*		
protein (Å^2^)	28.4	17.1
Bound compounds (Å^2^)	56.9 (Lys), 42.9 (Glu)	20.9 (HEPES)
solvent (Å^2^)	49.8	22.4
water (Å^2^)	36.8	15.2
*Ramachandran*		
*allowed (%)*	97.6	95.6
*favoured (%)*	2.4	4.4

Values in parentheses are for highest-resolution shell.

a *R*_meas_  =  ∑_hkl_ [N/(N−1)]^1/2^ ∑_i_ |I(hkl)−<I(hkl)> |/∑_hkl_ ∑_I_ I(hkl), where N is the multiplicity of a given reflection.

b *R*_cryst_  =  ∑||*F*_obs_| − |*F*_calc_||/∑|*F*_obs_| for all reflections.

c *R*_free_ was calculated on the 5% of data excluded from refinement.

### Fluorescence anisotropy measurements

Several H7 SRA oligoribonucleotides were ordered with a fluoro-uridine at the U206 equivalent position and a fluorescein probe at the 5′-end for the fluorescence anisotropy measurements (Dharmacon). Although we used a 5-fluorouridine (5-FU)-containing RNA, our experimental conditions did not generate a significant amount of covalently bound protein/RNA complexes (Figure S1). Steady-state fluorescence anisotropy measurements were performed in a 50 µL high precision fluorescence quartz cell (HellmaAnalytics, light pass 3×3 mm) with a Jobin-Yvon Horiba Fluoromax-4 spectrophotometer equipped with emission and excitation polariser filters. The samples were kept at 20°C with a VWR digital thermostat bath.

Anisotropy titrations were carried out by adding increasing amounts of FL hPus1p (or ΔhPus1p, or D146A ΔhPus1p) to a fixed concentration of Fluoroscein-RNA in 10 mM HEPES (pH 7.0) containing 150 mM NaCl, 5 mM MgCl_2_ and 5 mM TCEP. Binding constants were determined for a constant SRA concentration fixed at 1 µM. As Fluorescein was the fluorescent marker for these measurements, the excitation wavelength was adjusted to 490 nm, and the emission wavelength was fixed at 516 nm. The Scatchard equation was rewritten to fit the anisotropy, r, as follows:
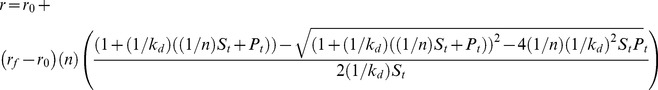
where P_t_ and S_t_ designate the total concentration of enzyme and Fluoroscein-SRA, respectively; r_f_ represents the anisotropy at the plateau when all the enzyme is bound; and r_0_ and r correspond to the anisotropy values of Fluoroscein-SRA in the absence and in the presence of a given concentration of enzyme, respectively. Values k_d_ and n correspond to the apparent dissociation constant and the number of binding sites of the enzyme for the SRA fragment, respectively. Titration curves were fitted with Microcal Origin 6.1 software based on the non-linear, least-squares method and the Levenberg–Marquardt algorithm. Each anisotropy data point has been measured four times in two independent experiments.

### RNA synthesis and pseudouridine formation assay

RNA substrates were synthesized using a sense T7 DNA oligonucleotide (5′TAATACGACTCACTATAG), an antisense template oligonucleotide (see below), and a high concentration of T7 RNA Polymerase, in the presence of 25 µCi [5-^3^H]-UTP (23.9 Ci/mmol, Moravek) with 100 µM cold UTP and 1.0 mM non-labeled ATP, GTP, and CTP as previously described (Milligan and Uhlenbeck, 1989). The antisense template oligodeoxynucleotides used for RNA synthesis were: mouse mitochondrial tRNA^Asp^ (with mutations T31C, T32C, and A39G), 5′TAAGATATATAGATTATTGATCTATAATTTAACCTTGACAGGGTTATGTAATTGATTTTACTAATATCTTCTATAGTGAGTCGTATTA; full-length H7 SRA, 5′CCCACAGGTGGGGACCTGGGAGCCTTACTTGAAGGAGGTGGAGGCCCCATTGGGGCTATAGTGAGTCGTATTA; truncation1 of H7 SRA, 5′CCCACAGGTGGGGACCTCGAAAGGCCCCATTGGGGCTATAGTGAGTCGTATTA; truncation 2 of H7 SRA,5′CCCACAGGTGGGGCGAACCCCATTGGGGCTATAGTGAGTCGTATTA; truncation 3 of H7 SRA, 5′CACAGGTGGGGCGAACCCATTGGCTATAGTGAGTCGTATTA; truncation 4 of H7 SRA, 5′CCCACAGGTATTGGGGCTATAGTGAGTCGTATTA.

The H7 SRA substrates were heated in water to 95°C for 2 minutes, allowed to cool slowly to 25°C (1 hour 15 minutes, ambient temperature 22–23°C), and then held at 25°C for 5 minutes. Substrate reactions (100 µl) with human pseudouridine synthases or β-galactosidase (3.2 µg protein per assay) were 50 mM ammonium chloride, 5 mM DTT, 25 mM Tris (pH 7.5) and 5 mM MgCl_2_, with the annealed RNA added to the other components at 25°C. Three separate assays were performed for each protein and RNA substrate combination. Incubations were held at 25°C for 4 hours, and then mixed with 500 µl Norit A charcoal (15% (w/v) in 0.2 N HCl), mixed vigorously, and then incubated at 37°C for 30 minutes with intermittent shaking. The charcoal was pelleted (16,000×g, 1.5 min) and the supernatant was filtered through a SPIN-X filter unit (Costar, Cambridge, MA) for 2 min at 8,000×g. The filtrate was mixed with 10 ml of scintillation fluid, and counted for 10 minutes [31], [32].

## Results and Discussion

**Figure 1:**
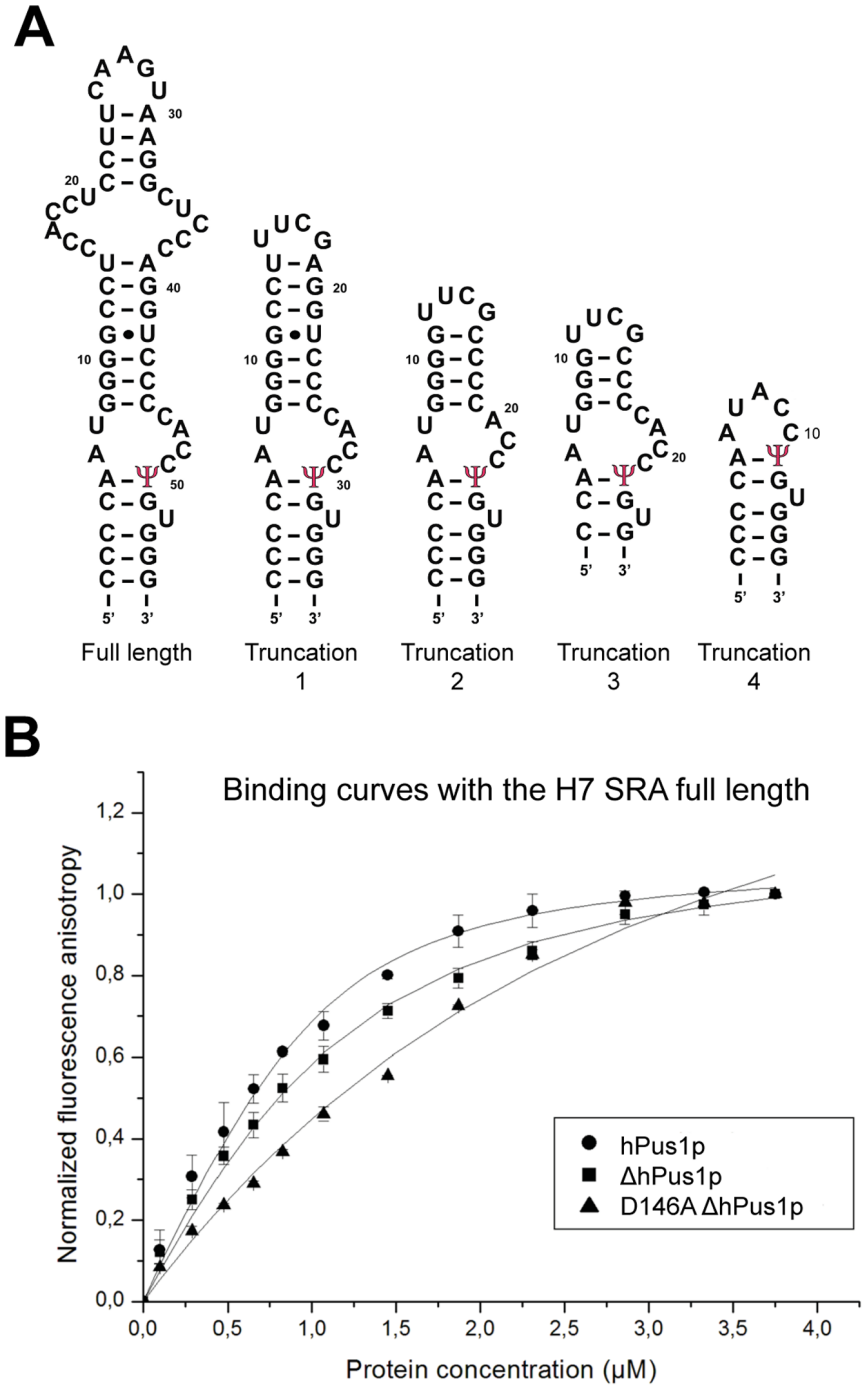
Human pseudouridine synthase 1 binds to various SRA constructs. (A) Secondary structures and sequences of the SRA constructs used in the binding tests with the putative U to Ψ position indicated. (B) Fluorescence anisotropy measurement of SRA binding to different hPus1p constructs. Fluorescein-conjugated SRA sequences were incubated with increasing amounts of the indicated proteins (full length hPus1, filled circle; ΔhPus1p, filled square; ΔhPus1p D146A, filled triangle). Normalized anisotropy values from 2–3 experiments were plotted against protein concentrations to measure binding affinities.

The hPus1p enzyme was shown to modify the non-coding SRA in a complex post-transcriptional mechanism, which regulates nuclear receptor-induced transactivation [12]. Recently the secondary structure of the SRA was determined [33]. The previously described SRA substructure STR5 was redefined and named H7 [12], [33]. Moreover, it was shown to harbour a slightly different structural organisation compared with the initial prediction [33]. In particular, the loop containing the targeted U (position 236 according to the 5′-end used in [33]) is asymmetric. We have used the latest H7 structural organisation to design various H7 substructures to investigate hPus1p/SRA interactions (Figure 1A).

### FL and ΔhPus1p enzymes are able to bind SRA substrates

First, we determined the binding stoichiometry and the binding affinities for three different hPus1p constructs using the fluorescein-labelled H7 RNAs (Figure 1B). We have used the human full-length enzyme (FL hPus1p), ΔhPus1p or ΔhPus1p D146A. The D146A mutated protein was further shown to behave as the WT enzyme in solution (Figure S1). FL hPus1p and the truncated forms of hPus1p bind to the various RNA constructs with a stoichiometry between 0.7 and 1 (Table 2) indicating that human Pus1p binds the H7 SRA as a monomer. This was also consistent with multiple angle light scattering measurements performed on ΔhPus1p (data not shown). This result was somewhat surprising since the bacterial TruA enzyme was shown to function as a dimer and crystallised as a dimer [6]. This also opens the question of how hPus1p binds to its substrate RNAs (tRNA or another class of RNA). It was shown previously that both molecules of the TruA dimer contact the tRNA substrate [34]. It therefore suggests that eukaryotic Pus1p enzymes will not recognise the elbow region of their tRNA substrates since these enzymes bind as a monomer, in contrast to the bacterial TruA enzymes [34]. Moreover, as observed for the human enzyme, yeast Pus1p is also active as a monomer [35]. The fact that the hPus1p enzyme acts as a monomer reflects the general properties of PUS enzymes from the other families, which were also shown to function as monomers [5], [7], [8], [9], [10], [36].

**Table 2:** Affinity constants between hPus1p and the various H7 SRA substrates.

Protein/RNA Complexes	Kd (nM)	SD^a^	n^b^	R^2^
hPus1p/H7 SRA FL	219	41	1	0.994
hPus1p/H7 SRA truncation 1	103	26	1	0.993
hPus1p/H7 SRA truncation 2	111	24	0.8	0.994
hPus1p/H7 SRA truncation 3	122	45	1	0.982
hPus1p/H7 SRA truncation 4	96	49	0.7	0.95
ΔhPus1p/H7 SRA FL	484	73	1	0.993
ΔhPus1p/H7 SRA truncation 1	219	33	0.8	0.994
ΔhPus1p/H7 SRA truncation 2	94	14	1	0.989
ΔhPus1p/H7 SRA truncation 3	95	21	0.7	0.992
ΔhPus1p/H7 SRA truncation 4	74	13	0.8	0.998
D146A ΔhPus1p/H7 SRA FL	2200	44	1	0.991
D146A ΔhPus1p/H7 SRA truncation 2	3900	65	1	0.995

a Listed are the standard deviation (SD) of two distinct binding experiments.

b n represents the number of binding sites between the protein and the SRA fragment.

R^2^ represents the coefficient of determination calculated for each fitting curve.

The measured binding affinities range from 100 to 500 nM indicating that hPus1p recognises the H7 SRA sequence with an affinity similar to its reported affinity for tRNAs (Table 2; Figure S2; [21]). Furthermore, we observe that the D146A mutation leads to a 10-fold decrease in the affinity of the enzyme for the full-length H7 substrate (K_d_ of 2.2 µM instead of 0.2 µM seen with the WT enzyme) clearly indicating that removing the catalytic aspartate destabilises the complex formation. Previous studies have stressed the importance of the catalytic D146 for the enzymatic activity of hPus1p [12], [37]. Furthermore, the K_d_ value of the mutant enzyme is comparable to the K_d_ measured for PUS enzymes from other subfamilies carrying the same homologous mutation at the active site (Table 2; [38]). It therefore indicates that the absence of the aspartate significantly destabilises complex formation, which is surprising since the proposed enzymatic role of the aspartate is to attack the C6 position of the uridine rather than stabilise the bound RNA [39].

Truncated hPus1p does have slight differences in affinity with H7 SRA substrates compared with the FL hPus1p (Table 2). Dissociation constants measured between FL hPus1p and the various H7 sequences are either equal or two-fold lower than the ones seen with ΔhPus1p, revealing that the truncated N- and C-terminal sequences are not essential for the stable association with the H7 RNA constructs (Figure 1B and Table 2). In detail, truncation 1 corresponds to the removal of distant sequences from the H7 stem loop (Figure 1A). The missing nucleotide sequence does not prevent stable complex formation, clearly indicating that hPus1p association seems to be restricted to the region closer to the uridine previously identified as the site of modification by hPus1p [15]. Therefore, association of hPus1p with H7 SRA is similar to its association with tRNA since it was shown previously that removing distal parts of the tRNA, such as the acceptor stem, did not impair association [21]. To further refine the hPus1p binding site on the H7 SRA, we generated the H7 truncations 2, 3, and 4. These three sequences are apparently recognised by both hPus1p proteins equally (Table 2). Therefore, we conclude that hPus1p only binds the nucleotide region surrounding either side of the targeted uridine (Figure 1A). This corroborates previous findings that RNA sequence immediately upstream or downstream of the targeted uridine does not significantly influence the overall affinity of hPus1p [21], [37].

### Pseudouridine synthase assays of hPus1p enzymes tRNA and HP7 SRA RNA substrates

The activities of the three hPus1p constructs (FL hPus1p, ΔhPus1p, and D146A ΔhPus1p) on the same H7 SRA substrates were assayed by the H^3^-release method (Table 3; [31], [32]). For these experiments, we used mouse mitochondrial tRNA^Asp^ as a positive control for hPus1p activity [22]. The FL hPus1p is highly active towards tRNA^Asp^, with significant levels of pseudouridine formation: while the ΔhPus1p has lower, but reproducible activity clearly indicating that the deleted N- and C-termini are not essential for either the RNA binding or the activity of the hPus1p enzyme (Tables 2 and 3). As expected, no activity could be measured with D146A ΔhPus1p with the catalytic aspartate residue 146 mutated (Table 3). The level of pseudouridine formation by the FL enzyme observed with several of the H7 SRA substrates is significant, although lower than for the tRNA (Table 3). It seems likely that this lower level of activity is due to the atypical H7 secondary structure. Indeed, the H7 RNA is composed of multiple stem loops but does not contain any four-way junctions as found in tRNAs. Recent studies of the hPus1p enzymatic activity using tRNA-derived substrates have shown the importance of having a stem loop 3′ to the modified uridine [21]. This requirement does not seem to be critical with the SRA modification (Figure 1A). The H7 SRA full-length substrate cannot be modified significantly by any of the hPus1p constructs. This observation may indicate that FL H7 SRA is not folded as predicted, possibly due to the absence of the remaining SRA sequence. Similar observations have been made for other large RNA molecules, such as group I introns, which form peripheral interactions essential for their functionality or recognition [40]. Truncation 1 is modified in a similar way by either FL or ΔhPus1p, which clearly indicates that the region between nucleotide 16 and 39 is not essential for both the association and the modification of the SRA by hPus1p (Tables 2 and 3). However, shortening the SRA construct further, from either side of the targeted uridine (truncation 2, 3, and 4), reduces the activity to background levels, although hPus1p can still form a stable complex with these sequences (Tables 2 and 3). These activity experiments suggest the importance of stem stability for protein-RNA recognition, and indicate that hPus1p requires stable stem structures on both sides of the previously identified uridine 236 of the SRA (Figure 1A and Table 3; [15]). These data complete previous observations regarding tRNA modification by hPus1p [21]. In this case, the minimal substrate required a stem 3′ to the modified uridine [21]. It is now clearly established that hPus1p has a relaxed sequence specificity, and that stems surrounding the targeted uridine play a non-essential role in the efficiency of pseudouridylation [13], [21], [41].

**Table 3:** Pseudouridine incorporation measured with various hPus1p enzymes.

	RNA substrates					
Enzyme^a^	tRNA^Asp^	H7 SRA full length	H7 SRA truncation 1	H7 SRA truncation 2	H7 SRA truncation 3	H7 SRA truncation 4
FL hPus1p	0.46 (0.01)	0.02 (<0.01)	0.12 (<0.01)	0.01 (<0.01)	0.05 (<0.01)	0.02 (<0.01)
ΔhPus1p	0.08 (0.01)	0.00 (<0.01)	0.10 (0.01)	0.01 (<0.01)	0.01 (0.02)	0.02 (0.02)
D146A ΔhPus1p	0.01 (<0.01)	0.00 (<0.01)	0.00 (<0.01)	ND	0.00 (<0.01)	ND

a The activities are in moles ψ/moles RNA with the mean of three separate assays and standard deviation (SD). With all of the substrates the maximum activity should be ∼1.0 moles ψ/moles RNA. Background levels of activity seen with Lac (β-galactosidase) have been subtracted. ND indicates that the combination of D146A ΔhPus1p D146A and the corresponding RNA substrate was not determined.

### Structural insights into the catalytic domain of the human Pus1p enzyme

**Figure 2:**
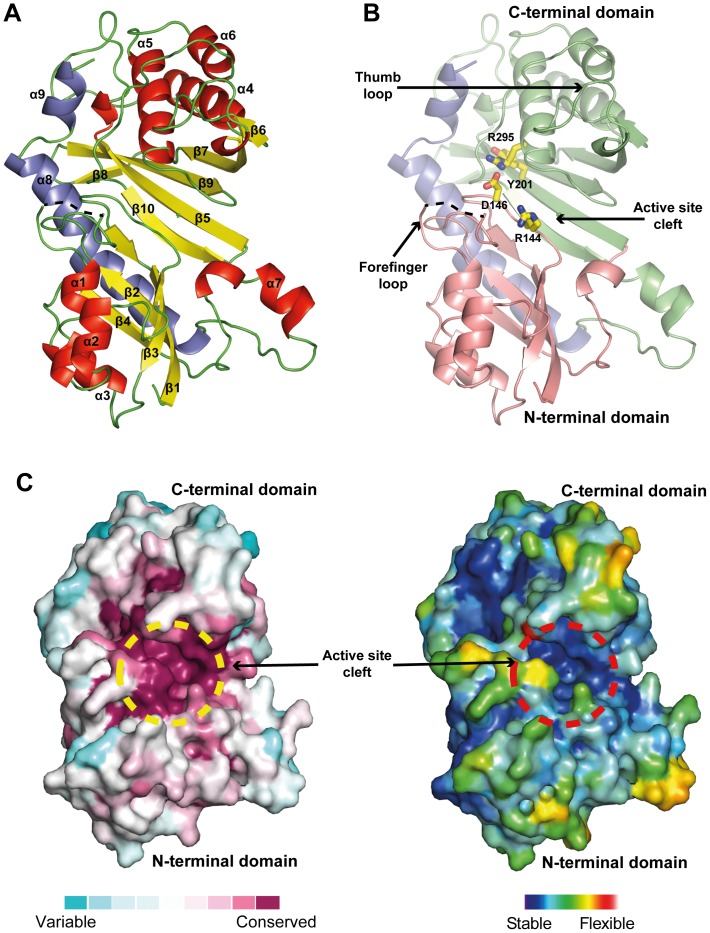
Overall views of the crystal structure of ΔhPus1p. (A) The ΔhPus1p monomer is shown in a cartoon representation and coloured according to secondary structure elements (red, α-helices; yellow, β-sheet; and green, loops). The C-terminal helices, which are specific to the hPus1p protein, are shown in blue. (B) Domain organization of ΔhPus1p. The N-terminal and C-terminal domains are orange and green, respectively; specific C-terminal helices are blue. The two domains form a cavity, which contains the active site. Two functionally important loops, the Forefinger and the Thumb loop are labelled. The catalytic amino acid residues are shown as sticks and coloured according to atom type (carbon, nitrogen, oxygen, and sulphur are yellow, blue, red, and gold, respectively) (C) Surface conservation and temperature factor representation of hPus1p. Conservation scores were determined with the ConSurf server ([42]), and plotted on the surface of the ΔhPus1p structure (blue, variable; red, conserved,). The temperature factors of the refined model were plotted by the same method. Low B factors (stable) are blue and high B factors (flexible) are red. A dashed circle (yellow or red) indicates the location of the enzyme active site.

The crystal structures of the ΔhPus1p and the D146A ΔhPus1p enzymes have been determined in complex with several molecules present in the crystallisation conditions. Crystals of ΔhPus1p belong to space group P2_1_2_1_2_1_, with two molecules per asymmetric unit. The structure was refined to 2.0 Å resolution, with an *R*_work_ of 18.4% and an *R*_free_ of 22.4% (Table 1; Figure 2A). Crystals of hPus1p D146A belong to space group P22_1_2_1_, and contain one molecule per asymmetric unit. The structure was refined to 2.7 Å resolution (*R*_work_ of 18.3% and *R*_free_ of 23.8%; Figure S3). The two hPus1p structures are essentially identical (Figure 2A and Figure S3). In both crystal structures, electron density for residues corresponding to the so-called forefinger loop (region 100–110) is absent, and so these residues have been omitted from the final structure (Figure 2B). A similar situation was reported recently by Czudnochowski and colleagues [28]. The high flexibility of this loop may be due to the absence of an RNA substrate, as this region is contacted by the tRNA in the TruB/tRNA complex structure [10], [17]. The region preceding the C-terminal helices of hPus1p (residues 348–352) is described by weak electron density, and so this region was built in molecule B of the ΔhPus1p structure only (Figure 2B).

The atomic model shows the typical fold for a pseudouridine synthase from the TruA family (Figure 2A and B; [6], [28], [41]). However, the ΔhPus1p enzyme has the peculiarity of having a C-terminal extension of approximately 35 residues, which folds on the face opposite to the active site cavity (Figure 2B). This extension covers residues involved in the dimerization of the bacterial TruA enzyme [6], [34], [41]. If we superimpose our structure of the ΔhPus1p enzyme onto the bacterial TruA dimer, it clearly shows that the mammalian enzyme cannot form a dimer (Figure S4). Therefore, this structure provides further evidence that the mammalian Pus1p enzyme functions as a monomer (Figure 2B). Conservation analysis, using the ConSurf server [42] fed with a multiple alignment done by the program PipeAlign [43], shows that the catalytic core is highly conserved (Figure 2C). Similarly, plotting temperature factors of the atomic model also indicates that the active site as the most stable region of the protein (Figure 2C). Furthermore, we observe that the most conserved and stable regions of the ΔhPus1p structure are formed by the loops surrounding the catalytic site, suggesting that this cavity exhibits plasticity, possibly to accommodate the various RNA substrates (Figure 2C). We also notice that the additional C-terminal helix, α8, is poorly conserved at the sequence level, although all eukaryotic pseudouridine synthase sequences have a predicted helical extension at this location. It is likely that such conservation of a secondary structural element reflects the functional importance of the helix. One possible role would be to stabilise the two N- and C-terminal sub-domains of hPus1p (Figure 2B). The helix may make hPus1p more rigid so that it behaves as a platform for RNA modification with only minor active site adaptations to the incoming RNA substrates.

**Figure 3:**
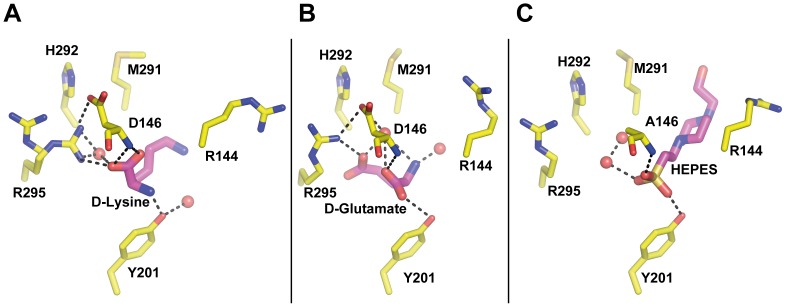
Close view of the active site residues from the ΔhPus1p and the ΔhPus1p D146A crystal structures. (A) A D-lysine molecule from the crystallisation condition is bound within the active site of molecule A of the ΔhPus1p enzyme. (B) A D-glutamate molecule is bound in the active site of molecule B of the same crystal structure. (C) A HEPES molecule is found in the active site of the inactive ΔhPus1p D146A crystal structure. Amino acids in the active site are shown and coloured as in Figure 2B. Bound molecules are shown as sticks and coloured according to atom type (carbon, nitrogen, oxygen, and sulphur are magenta, blue, red, and gold, respectively). Water molecules are shown as red spheres. Hydrogen bonds between the amino acids and the protein atoms are indicated as black dash lines.

Differences between structures of ΔhPus1p and ΔhPus1p D146A are limited to external, poorly conserved loops, which confirm the flexibility of these regions previously identified by Czudnochowski et al. (Figure 2C; Figure S3; [28]). Extensive co-crystallisation assays with small SRA substrates or uridine derivatives resulted in diffracting crystals although the corresponding electron density maps did not show any extra density peaks near the active site of ΔhPus1p (results not shown). However, in the presence of the amino acid mixture from the crystallisation conditions, the *F*_obs_-*F*_calc_ and the 2*F*_obs_-*F*_calc_ Fourier electron density maps systematically showed strong connected electron density near the active site residues D146, Y201, H292, and R295 (Figure 3). These densities were fitted with the various compounds present in the crystallisation conditions, which allowed us to identify unambiguously the atypical molecules bound in the active site (Figure 3 and Figure S5). Three different molecules were bound: i) a lysine in molecule A of the ΔhPus1p crystal. ii) a glutamate in molecule B of the ΔhPus1p crystal. iii) a HEPES molecule in the D146A ΔhPus1p crystal (Figure S6).

In molecule A, the carboxyl group of the lysine molecule forms a hydrogen bond with the backbone atoms of residue D146, while the lysine amide group is bound to the hydroxyl group of residue Y201 (Figure 3A). In molecule B of the same crystal form, the backbone of the glutamate compound is hydrogen bonded to the hydroxyl group of residue Y201, while the main chain and the side chain of residue R295 stabilise the OE1 and OE2 positions of the glutamate (Figure 3B). The various interactions between these two compounds and the backbone atoms of hPus1p are reminiscent to the one established between the pseudouridine nucleotide and the TruB protein [10], [17]. The crystal structures presented here indicate that the active site of ΔhPus1p can accommodate several types of molecule through the establishment of multiple hydrogen bonds. The carboxyl groups of the two amino acids are located near the backbone of residue D146 and occupy roughly the same position in both molecules of ΔhPus1p (Figure 3A and 3B). However, the glutamate and the lysine side chains are orientated in opposite directions. The side chain of the glutamate points towards residue R295 of α5, while the lysine NZ atom faces residue R144 of the catalytic loop. None of these bound molecules form hydrogen bonds with the catalytic aspartic acid D146 side chain. The presence of these two compounds with opposite ionic characteristics, i.e. positively or negatively charged, suggests that the active site cavity of hPus1p may mimic a dipole. We speculate that such dipolar properties could facilitate the necessary rotation of the pyrimidine ring during catalysis.

A HEPES molecule is bound in the active site of the D146A mutant structure (Figure 3C) and is located at the same position as the MES compound found in the crystal structure of Pus1p solved recently (PDB id 4ITS; [28]). The HEPES molecule is bound in the active site, and its sulphate group occupies exactly the same orientation as the sulphate from the MES molecule (Figure 3C and Figure S6). Similarly, N4 of the MES and N1 of the HEPES molecule are nearly indistinguishable (Figure 3C and Figure S6). A HEPES molecule is bound to the active site in the structure of pseudouridine synthase 10, although its orientation was different (Figure S6). The NZ atom of the bound lysine is also located within the same area, which could indicate that ΔhPus1p has a preferential site for nitrogen stabilisation near the residue D146 - the residue initiating the pseudouridine reaction (Figure 3C). In a superposition of the Leucyl tRNA-bound TruA (PDB id 2NR0; [34]) and the ΔhPus1p D146A structures, the N9 atom of the flipped guanosine is nearly co-incident with the HEPES molecule (Figure S6), reinforcing the proposed electropositive region near the active site residue D146. The piperazine moiety of the HEPES compound superimposes with the ribose ring of the guanosine (Figure S6). The positioning of a sulphate group inside the active site of ΔhPus1p may be irrelevant for the enzymatic reaction *per se*, but clearly indicates that the active site cavity is not neutral, a property that was suggested to be important during the catalytic cycle (Figure 3A and 3B).

## Conclusion

The structures presented here further demonstrate the unusual capacity of hPus1p to modify various types of RNA substrates. These atomic models also indicate that hPus1p functions as a monomer in contrast to its bacterial counterpart, TruA. We suggest that the large C-terminal extension, which folds into several α-helices, prevents the dimerization of hPus1p. This monomeric behaviour may well explain the diversity of substrates that are recognised by the enzyme, since we show that a substrate with a large asymmetric loop, preceded and followed by stable stems, may be targeted by hPus1p. Our crystallographic models contain various small molecules bound to the hPus1p active site, which, we believe, may reflect its unexpected electronic properties. Further structural characterisation of hPus1p complexed with SRA will be necessary to compare the specific determinants behind the modification of this ncRNA.

## Supporting Information

Figure S1 **Size exclusion chromatography of the purified WT and D146A hPus1p.** Following their expression and purification, both proteins were loaded on a superdex200 gel filtration column. The WT and the D146A hPus1p are shown as a blue and a red trace respectively.
(TIF)

Figure S2 **Denaturing gel analysis showing the absence of covalent 5-FU-RNA/hPus1p complex being formed during our binding experiments.** 5-FU modified RNA truncation 1 (at 36 µM) and the hPus1p (at 20 µM) were incubated for 3 h at the indicated temperature. Denaturing buffer was then added and the samples were either directly loaded or incubated for 5 min at 95°C before loading in a 12% SDS-PAGE.
(TIF)

Figure S3 **Overall view of the ΔhPus1p atomic models.** (A) Structure of the D146A ΔhPus1p. The protein is shown as a cartoon and colored as follow: orange, N-terminal domain; green, C-terminal domain; blue, for the C-terminal helices. Amino acids important for the catalytic cycle are shown as sticks and coloured according to atom type (carbon, yellow; nitrogen, blue; oxygen, red). (B) The structures of the two ΔhPus1p monomers and the D146A ΔhPus1p monomer are superimposed and coloured in orange, blue and white respectively.
(TIF)

Figure S4 **Dimerization of the Pus1 enzymes.** (A) The dimer of the bacterial TruA enzyme is shown as observed in the crystal structure of the E. coli TruA enzyme (PDB code 2NR0; [34]). (B) Our atomic model of the catalytic domain of the hPus1p has been superimposed onto each TruA molecule (coloured in orange and green). The C-terminal helices are clearly clashing with each other and therefore would prevent such multimerisation (coloured in cyan and blue). Protein structures are shown as cartoon in both panels.
(TIF)

Figure S5 **Difference Fourier electron density maps found in the active sites of each ΔhPus1p molecule.** The left column shows the F_o_-F_c_ electron density map contoured at 2.7 σ for the molecule A. The right column shows the difference electron density map found in the molecule B contoured at the same level. Individual amino acids present in the crystallization buffer were fitted into the densities. Each compound is shown as stick colored according to atom type (carbon, gray or purple; nitrogen, blue; oxygen, red). The chosen amino acid for each ΔhPus1p molecule is colored in purple. The difference electron density map was calculated using the protein only as a model and are shown as green mesh.
(TIF)

Figure S6 **Superposition of the molecules found in the active site of various Pseudouridine Synthase 1 structures.** (A) Superposition of the D146A ΔhPus1p atomic model bound to an HEPES molecule with the catalytic domain of the hPus1p structure bound to a MES compound (shown as sticks and colored in blue for the protein and white for the HEPES; [28]). (B) Superposition of the D146A ΔhPus1p atomic model with the structure of the human PUS10 enzyme also bound to an HEPES compound (shown as stick and colored in orange for the protein and white for the HEPES; PDB code 2V9K; [5]). Amino acids from the PUS10 enzyme are labelled in italic. The HEPES has an opposite orientation with the phosphate outside the active site of PUS10. (C) Superposition of the D146A ΔhPus1p atomic model with the structure of the E. coli TruA enzyme in complex with tRNA^leu^ (shown as stick and colored in green for the protein and white for the guanosine; PDB code 2NR0; [34]).
(TIF)
