# Correction: Steroid Receptor RNA Activator (SRA) Modification by the Human Pseudouridine Synthase 1 (hPus1p): RNA Binding, Activity, and Atomic Model

**DOI:** 10.1371/journal.pone.0122147

**Published:** 2015-03-25

**Authors:** The PLOS ONE Staff

The following information is missing from the Funding section: This work was also partially supported by the Worldwide Cancer Research grant (N°14-0346 to S.T.).
