# Supplementary figures and images for: Steroid Receptor RNA Activator (SRA) Modification by the Human Pseudouridine Synthase 1 (hPus1p): RNA Binding, Activity, and Atomic Model
Source: PLoS One. 2014 Apr 10;9(4):e94610. doi: 10.1371/journal.pone.0094610 (PMC3983220; doi:10.1371/journal.pone.0094610)

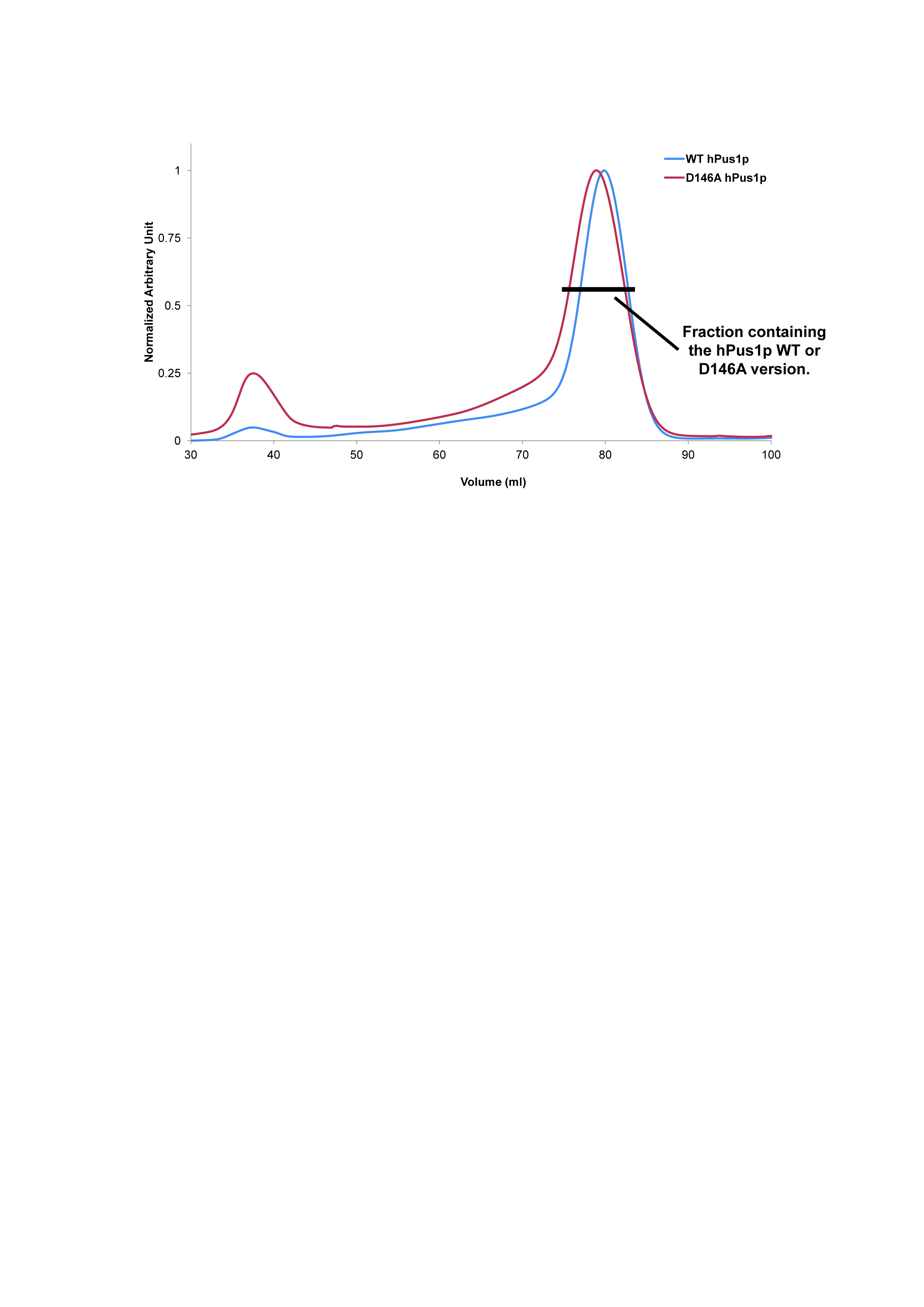

Supplement: Figure S1 — Size exclusion chromatography of the purified WT and D146A hPus1p. Following their expression and purification, both proteins were loaded on a superdex200 gel filtration column. The WT and the D146A hPus1p are shown as a blue and a red trace respectively. (TIF) [file pone.0094610.s001.tif]

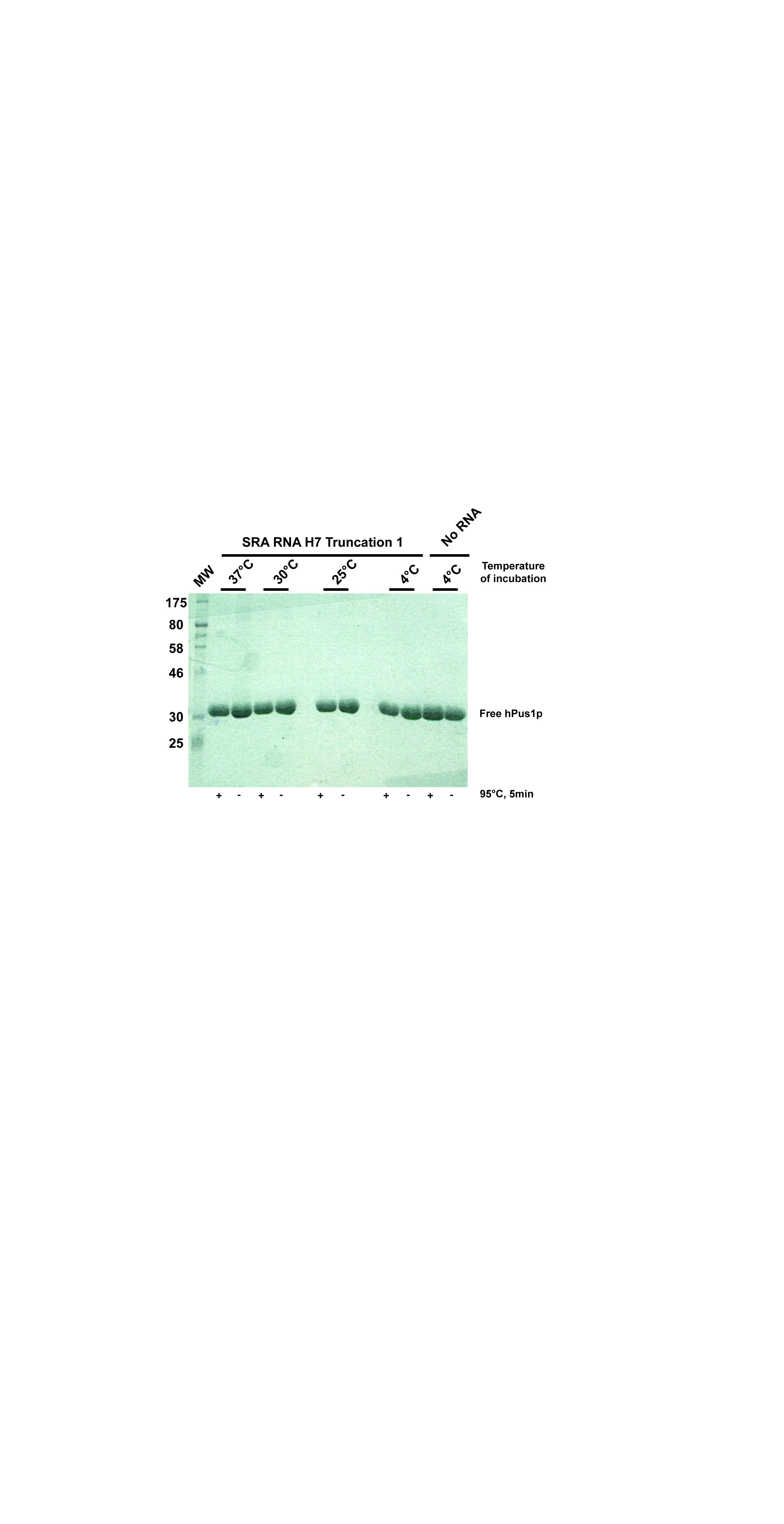

Supplement: Figure S2 — Denaturing gel analysis showing the absence of covalent 5-FU-RNA/hPus1p complex being formed during our binding experiments. 5-FU modified RNA truncation 1 (at 36 µM) and the hPus1p (at 20 µM) were incubated for 3 h at the indicated temperature. Denaturing buffer was then added and the samples were either directly loaded or incubated for 5 min at 95°C before loading in a 12% SDS-PAGE. (TIF) [file pone.0094610.s002.tif]

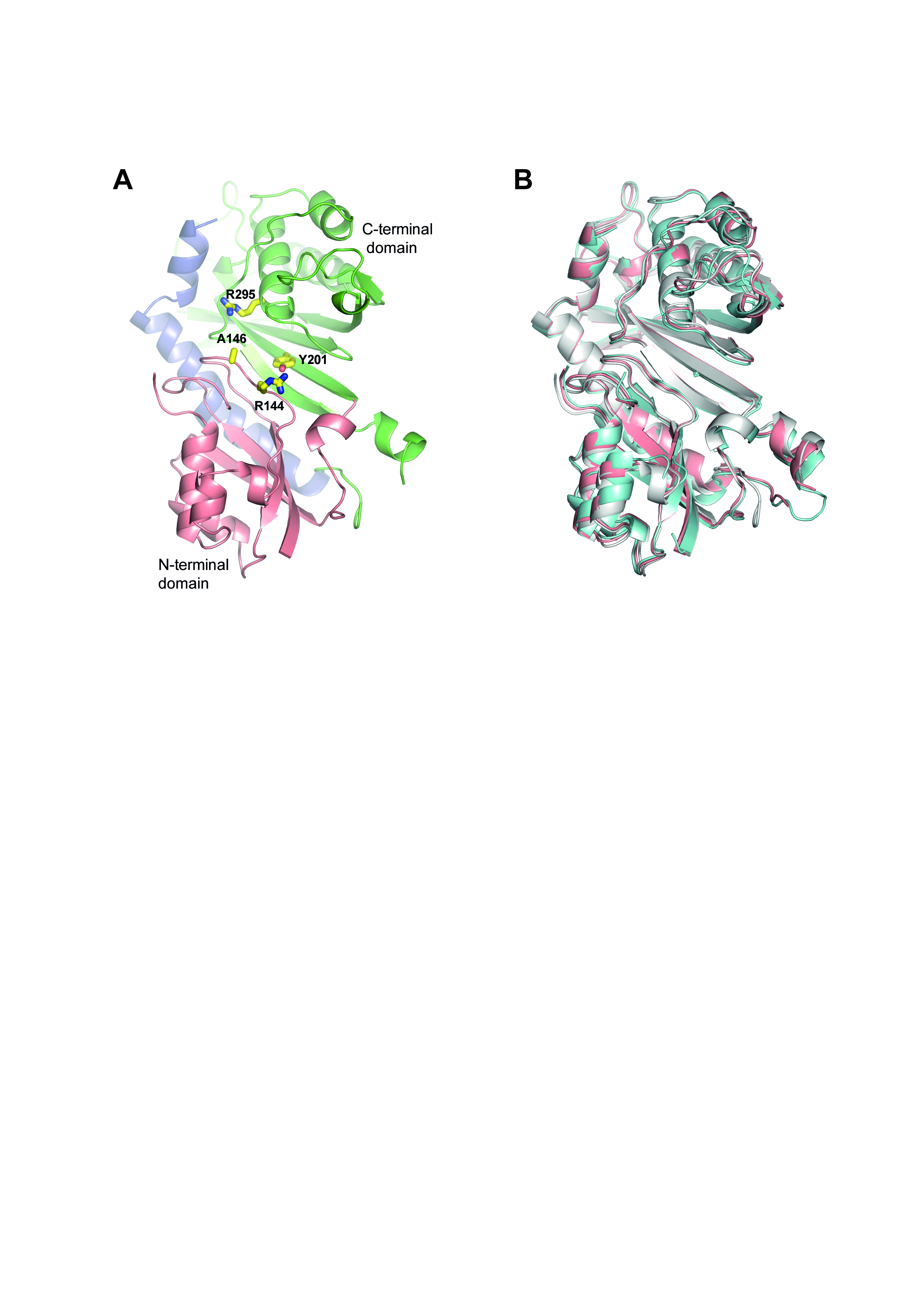

Supplement: Figure S3 — Overall view of the ΔhPus1p atomic models. (A) Structure of the D146A ΔhPus1p. The protein is shown as a cartoon and colored as follow: orange, N-terminal domain; green, C-terminal domain; blue, for the C-terminal helices. Amino acids important for the catalytic cycle are shown as sticks and coloured according to atom type (carbon, yellow; nitrogen, blue; oxygen, red). (B) The structures of the two ΔhPus1p monomers and the D146A ΔhPus1p monomer are superimposed and coloured in orange, blue and white respectively. (TIF) [file pone.0094610.s003.tif]

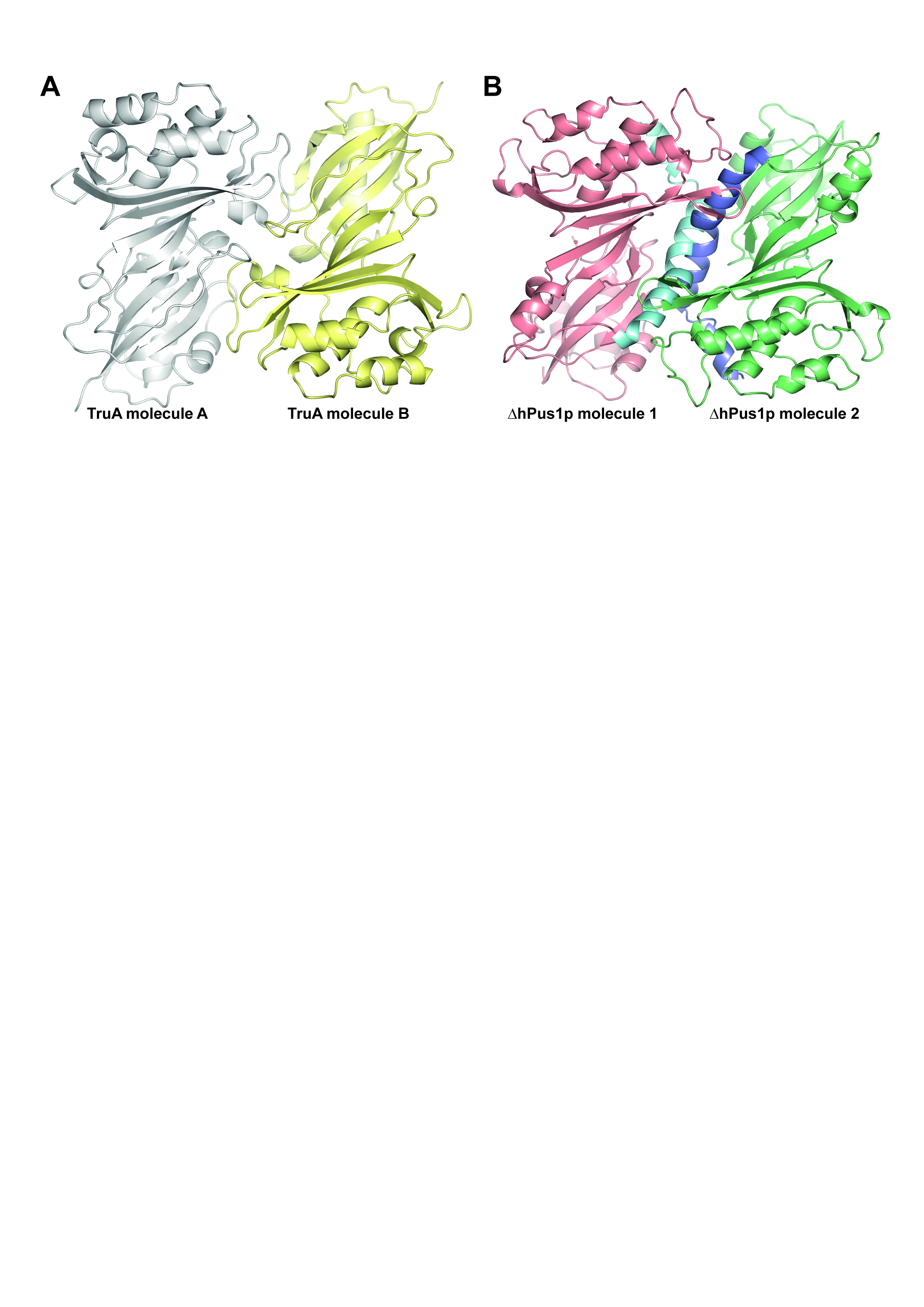

Supplement: Figure S4 — Dimerization of the Pus1 enzymes. (A) The dimer of the bacterial TruA enzyme is shown as observed in the crystal structure of the E. coli TruA enzyme (PDB code 2NR0; [34]). (B) Our atomic model of the catalytic domain of the hPus1p has been superimposed onto each TruA molecule (coloured in orange and green). The C-terminal helices are clearly clashing with each other and therefore would prevent such multimerisation (coloured in cyan and blue). Protein structures are shown as cartoon in both panels. (TIF) [file pone.0094610.s004.tif]

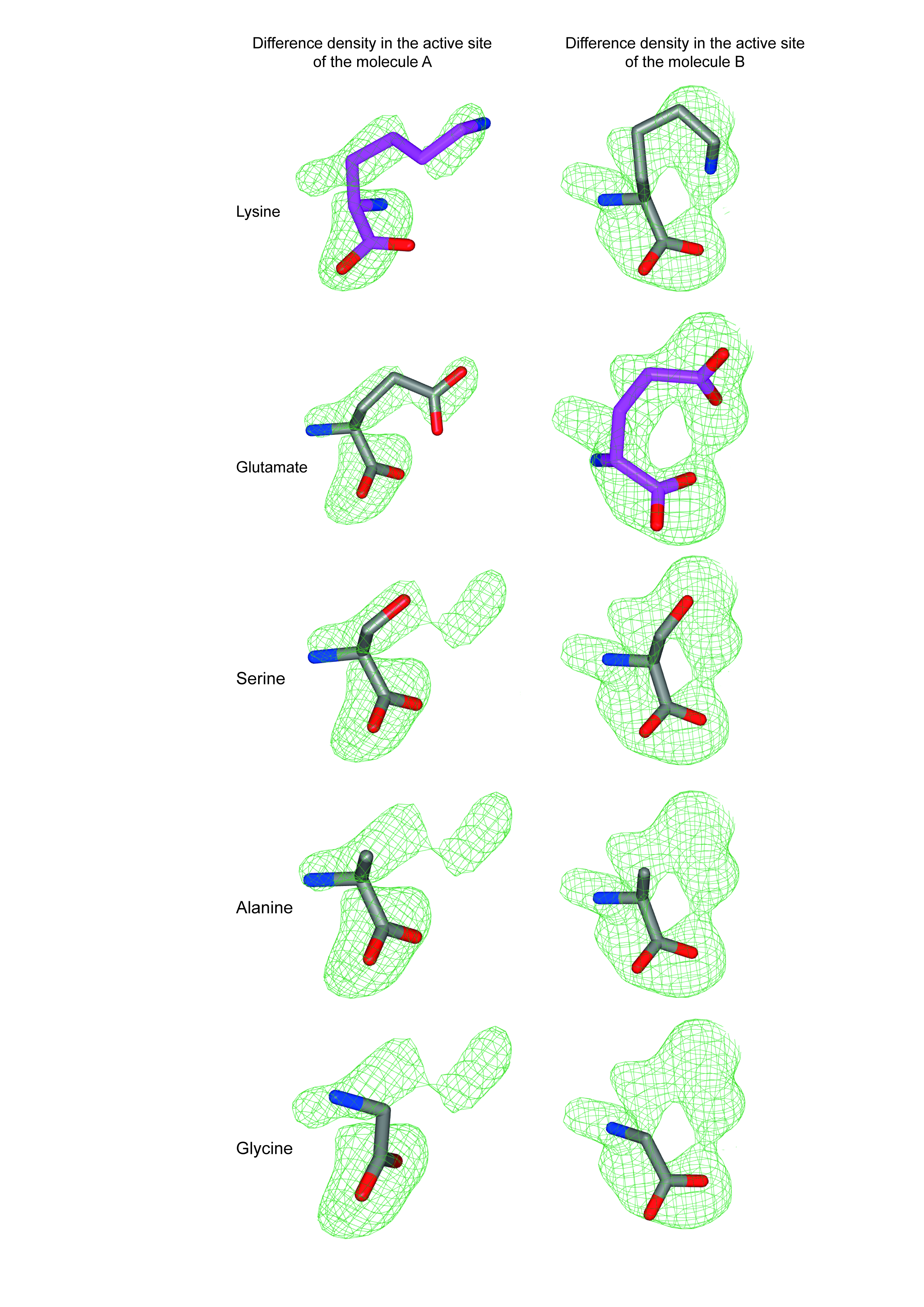

Supplement: Figure S5 — Difference Fourier electron density maps found in the active sites of each ΔhPus1p molecule. The left column shows the Fo-Fc electron density map contoured at 2.7 σ for the molecule A. The right column shows the difference electron density map found in the molecule B contoured at the same level. Individual amino acids present in the crystallization buffer were fitted into the densities. Each compound is shown as stick colored according to atom type (carbon, gray or purple; nitrogen, blue; oxygen, red). The chosen amino acid for each ΔhPus1p molecule is colored in purple. The difference electron density map was calculated using the protein only as a model and are shown as green mesh. (TIF) [file pone.0094610.s005.tif]

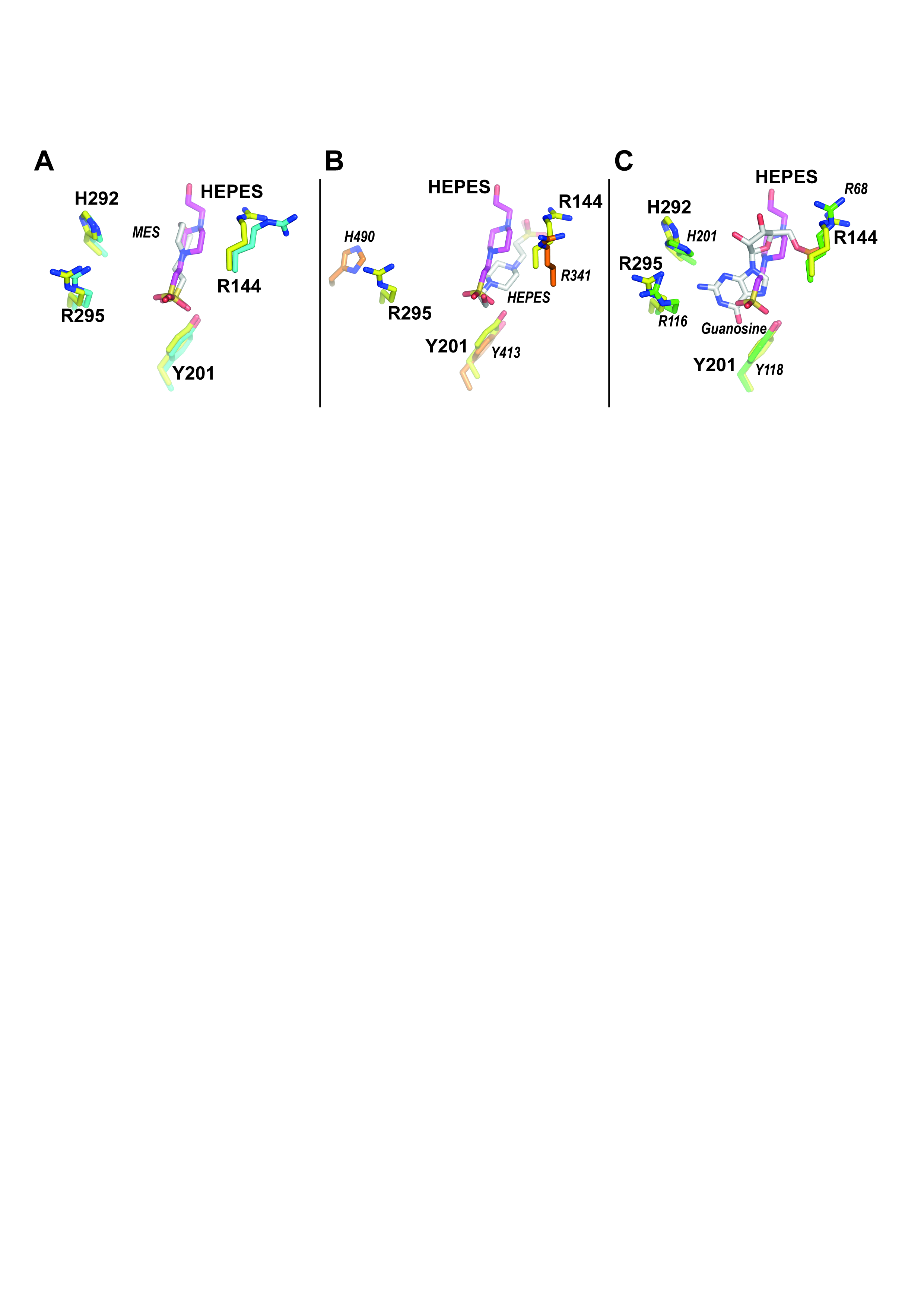

Supplement: Figure S6 — Superposition of the molecules found in the active site of various Pseudouridine Synthase 1 structures. (A) Superposition of the D146A ΔhPus1p atomic model bound to an HEPES molecule with the catalytic domain of the hPus1p structure bound to a MES compound (shown as sticks and colored in blue for the protein and white for the HEPES; [28]). (B) Superposition of the D146A ΔhPus1p atomic model with the structure of the human PUS10 enzyme also bound to an HEPES compound (shown as stick and colored in orange for the protein and white for the HEPES; PDB code 2V9K; [5]). Amino acids from the PUS10 enzyme are labelled in italic. The HEPES has an opposite orientation with the phosphate outside the active site of PUS10. (C) Superposition of the D146A ΔhPus1p atomic model with the structure of the E. coli TruA enzyme in complex with tRNAleu (shown as stick and colored in green for the protein and white for the guanosine; PDB code 2NR0; [34]). (TIF) [file pone.0094610.s006.tif]
